# Ebola Virus Transmission Initiated by Systemic Ebola Virus Disease Relapse

**DOI:** 10.1056/NEJMoa2024670

**Published:** 2021-04-01

**Authors:** Placide Mbala-Kingebeni, Catherine Pratt, Mbusa Mutafali Ruffin, Matthias G. Pauthner, Faustin Bile, Antoine Nkuba Ndaye, Allison Black, Eddy Kinganda Lusamaki, Martin Faye, Amuri Aziza, Moussa M Diagne, Daniel Mukadi, Bailey White, James Hadfield, Karthik Gangavarapu, Nella Bisento, Donatien Kazadi, Bibiche Nsunda, Marceline Akonga, Olivier Tshiani, John Misasi, Aurelie Ploquin, Victor Epaso, Emilia Sana Paka, Yannick Tutu Tshia N’kasar, Fabrice Mambu, Francois Edidi, Meris Matondo, Junior Bula Bula, Boubacar Diallo, Mory Keita, Marie Roseline Darnycka Belizaire, Ibrahima Soce Fall, Abdoulaye Yam, Mulangu Sabue, Anne W. Rimion, Elias Salfati, Ali Torkamani, Marc A. Suchard, Ian Crozier, Lisa Hensley, Andrew Rambaut, Ousmane Faye, Amadou Sall, Nancy J. Sullivan, Trevor Bedford, Kristian G. Andersen, Michael R. Wiley, Steve Ahuka-Mundeke, Jean-Jacques Muyembe Tamfum

**Affiliations:** 1 Institut National de Recherche Biomédicale, Kinshasa, DRC; 2 University of Kinshasa, Kinshasa, DRC; 3 University of Nebraska Medical Center, Omaha, NE, USA; 4 International Medical Corps, Los Angeles, CA, USA; 5 The Scripps Research Institute, La Jolla, CA, USA; 6 Ministère de la Sante, Kinshasa, DRC; 7 Fred Hutchinson Cancer Research Center, Seattle, WA, USA; 8 Institut Pasteur de Dakar, Dakar, Senegal; 9 Vaccine Research Center, National Institute of Allergy and Infectious Diseases (NIAID), National Institutes of Health (NIH), Bethesda, Maryland, USA; 10 World Health Organization, Geneva, CHE; 11 University of California, Los Angeles, CA, USA; 12 Clinical Monitoring Research Program Directorate, Frederick National Laboratory for Cancer Research, Frederick, MD, USA; 13 Integrated Research Facility at Fort Detrick, National Institute of Allergy and Infectious Diseases, National Institutes of Health, Frederick, MD, USA; 14 University of Edinburgh, Edinburgh, UK

## Abstract

During the 2018-2020 Nord Kivu Ebola virus disease (EVD) outbreak in the Democratic Republic of the Congo, an individual who had received the Merck rVSV-ZEBOV vaccine was diagnosed with EVD. His treatment included an Ebola virus-specific monoclonal antibody (mAb114), and he recovered within 14 days but re-presented six months later with severe EVD-like illness and Ebola virus viremia and died. We initiated an epidemiological and genomic investigation that showed the patient had a relapse of acute EVD, which led to a transmission chain that resulted in 91 cases spanning six health zones over four-months.

Human-to-human transmission of Ebola virus (EBOV) typically occurs through direct contact with infectious blood or bodily fluids.^1^ Though EBOV persistence has been well documented in EVD survivors, secondary transmission through contact with infectious bodily fluids (e.g. semen or breast milk) is relatively rare but has been linked to flare-up events.^2^ Meningoencephalitis and uveitis syndromes associated with infectious EBOV recovered from cerebrospinal fluid (CSF) and aqueous humor, respectively, have been documented in two EVD survivors; neither led to further transmission.^3,4^ Here, we report the relapse of acute EVD in an individual infected with the EBOV Ituri variant during the 2018-2020 Nord Kivu EVD outbreak in the Democratic Republic of the Congo (DRC)^5^, which led to onward transmission. His relapse occurred 149 days after discharge from an Ebola treatment unit (ETU) and sparked a transmission chain of 91 cases.

## Case Report

### Patient history and epidemiology

#### Initial EVD episode (June 2019)

**Figure 1:**
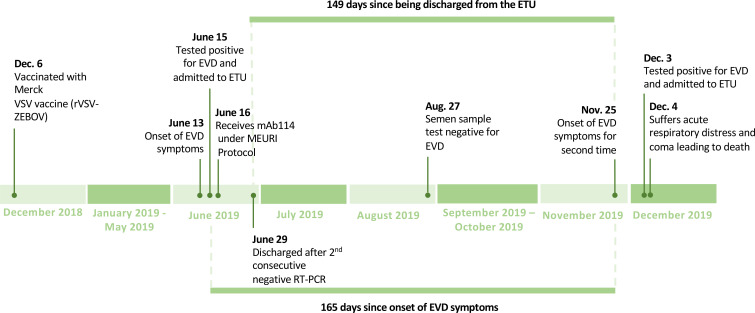
Timeline of the first and second EVD episodes.

A 25-year-old male motorcycle taxi driver presented to the ETU in Mangina, DRC, on June 15, 2019, with fever, nausea, vomiting, asthenia, anorexia, myalgia, and chest pain. According to the patient’s medical record, he received the rVSV-ZEBOV^6^ (Merck, NJ, USA, LOT WL00064825) vaccine six months prior, on December 6, 2018, due to being a contact of a confirmed EVD case. Despite his prior vaccination, we detected EBOV RNA in the patient's blood using the GeneXpert platform (Xpert Ebola Assay, Cepheid, CA, USA, **Table 1**) and diagnosed him with EVD. We administered the experimental mAb114 monoclonal antibody treatment^7^ starting on June 16, 2019, under the Monitored Emergency Use of Unregistered and Investigational Interventions (MEURI) protocol.^8^ In addition, we provided standard supportive care that included an antibiotic (intravenous ceftriaxone), antimalarial (artesunate-amodiaquine), a proton pump inhibitor (omeprazole), and magnesium supplementation. The patient was discharged from the ETU on June 29, 2019 after two consecutive negative PCR results (**Figure 1****,** **Table 1**). On August 27, 2019, a semen sample was collected under the national program monitoring EVD survivors and tested negative for EBOV RNA (**Table 1**). The patient did not follow up for additional semen testing.

**Table 1:** Ebola qRT-PCR and ELISA diagnostic test results for the first and second EVD episodes Gene Xpert (Cepheid) diagnostic quantitative RT-PCR Ct-value results for Ebola GP (ctGP) and Ebola NP40 (ctNP) are shown for indicated sample types and time points. Likewise, anti-Ebola GPIgG EC_50_ binding titers (Alpha Diagnostic International) are listed. Samples from which full viral genomes were determined are indicated in the rightmost column.

Sample ID	Lab ID	Date Sample Collected	Sample Type	Gene Xpert ctGP	Gene Xpert ctNP	Ebola GP IgG EC_50_ titer	Virus Sequenced
Sample d1	MAN4194	15-Jun-19	serum	32.5	29.9	neg	Yes
	MAN4337	18-Jun-19	serum	neg	41.7	-	-
	MAN4434	20-Jun-19	serum	41.3	39.2	-	-
	MAN4524	22-Jun-19	serum	neg	38.5	-	-
	MAN4694	25-Jun-19	serum	neg	38.0	-	-
	MAN4796	27-Jun-19	serum	neg	neg	-	-
Sample d14	MAN4907	29-Jun-19	serum	neg	neg	1:77,579	-
	-	27-Aug-19	semen	neg	neg	-	-
Sample d171	MAN12309	3-Dec-19	serum	33.3	30.1	1:164,609	Yes
Sample d173	MAN12369	5-Dec-19	swab	28.7	24.8	-	Yes

#### Second EVD episode and onward transmission (November 2019 - March 2020)

On November 25, 2019, 149 days after being discharged from the ETU, our patient experienced the onset of headache, asthenia, myalgia, polyarthralgia, and anorexia. He was seen at a local health center where he received unspecified treatment. On November 26, he developed abdominal pain, nausea, diarrhea, melena, chest pain, pain in the spine, jaundice, conjunctival injection, and epistaxis. The patient consulted a traditional practitioner and was hospitalized for two days, receiving unspecified treatment. After the symptoms increased in severity, community members alerted the EVD response team and eight days following the onset of illness (December 03, 2019), the patient was transferred to the ETU in Mangina. He had transient loss of consciousness soon after arrival. Initial clinical examination revealed a bedridden patient with pale palpebral conjunctiva, icteric bulbar conjunctiva, soft and depressible abdomen with epigastric tenderness, swelling of the left upper limb, and tender ecchymosis and bleeding at a venipuncture site. Vital signs at arrival included a heart rate of 91 bpm, respiratory frequency of 26 cycles / min, and blood pressure of 100/60 mmHg; the SpO2 was 99%. He was tested for HIV (Determine™, Abbott) and malaria using rapid diagnostic tests (RDT), with negative results. A blood sample on December 3, 2019 tested positive for EBOV RNA (**Table 1**), and he was diagnosed with EVD for the second time in six months.

He was treated with antibiotics (ceftriaxone, metronidazole, amoxicillin/clavulanate), antimalarial (artesunate-amodiaquine), a proton pump inhibitor (omeprazole), antiemetic (ondasetron) and rehydration fluid. On December 4, the patient's condition deteriorated with loss of consciousness, gingivorrhagia, anemia, and dyspnea associated with painful hepatomegaly on palpation. Clinical laboratories revealed acute kidney injury, liver injury (elevated hepatic transaminases, hyperbilirubunemia), hyponatremia, severe hypoalbuminemia, and a markedly elevated C-reactive protein, all consistent with multi-organ failure or dysfunction (**Table S1**). The patient was treated with oxygen and a blood transfusion. Despite the treatment, the patient developed acute respiratory distress and coma that led to death. A post-mortem oral swab on December 5, 2019, was positive for EBOV RNA (**Table 1**). An epidemiological investigation found the patient had directly infected 29 people while he was symptomatic in the community and visited local health clinics for treatment. Sixty-two additional cases resulted from onward transmission spanning six health zones over four months.

### Molecular and serological investigation

**Figure 2:**
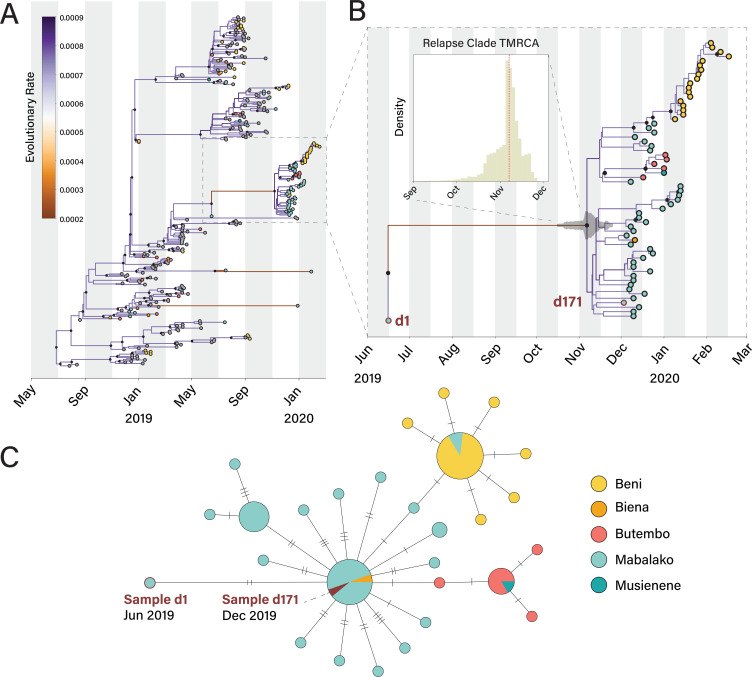
**Phylogenetic and epidemiological analysis of the relapse patient and linked cases A)** Maximum clade credibility tree with a two-rate clock model where branches indicating persistent infection were allowed to have a different rate of evolution from the rest of the tree. The tree was estimated using sequenced isolates with >95% coverage from the current Nord Kivu EBOV outbreak in DRC (n=297), colored by health zone. Branch colors indicate the evolutionary rate as indicated in substitutions/site/year. Internal nodes of the tree with a posterior probability > 50% are marked with black circles. **B)** Zoomed-in view of the time tree showing the first (Sample d1) and second EVD episodes (Sample d171) of the relapse patient, as well as 61 viral genomes sampled from epidemiologically linked cases. The 95% highest posterior density of the estimated time to most recent common ancestor (TMRCA) for the relapse clade is indicated in gray, and shown in full in the top left corner. The median TMRCA was estimated to be November 7th, 2019 (95% HPD: 15 Oct 2019 - 24 Nov 2019). The evolutionary rate between samples d1 and d7 is 4-fold reduced compared to the overall outbreak (see **Figure S2**). Data taken from https://nextstrain.org/community/inrb-drc/ebola-nord-kivu and released on NCBI GenBank database. **C)** Haplotype network of the relapse case patient and 72 epidemiology linked cases across five different health zones in DRC. Circle sizes represent the number of cases.

A genomic investigation was launched to support the epidemiological findings and differentiate between relapse and reinfection, i.e. recurrence of his initial disease from June 2019 versus reinfection from an active transmission chain during November 2019. We sequenced diagnostic samples from the patient’s first infection (blood sample from June 15, 2019 [d1]) and second infection (blood sample from December 3, 2019 [d171] and post-mortem oral swab [d173]), along with 72 epidemiologically linked (epi-linked) cases (**Table S2**). Our comparison of these data to previously sequenced Nord Kivu outbreak samples revealed that all of our patient’s samples (d1, d171, and d173) and the 72 epi-linked cases shared a unique mutation (G6800A), which separated these sequences from the rest of the outbreak (**Figure S1A**). We found that the samples taken during the second infection (d171 and d173), along with the 72 epi-linked cases, shared two unique mutations (T5578C/non-coding and A6867G/E280G GP) that genetically linked the cluster and placed the d1 sequence ancestral to the relapse cluster (**Figure 2C****, Figure S1B**).

We used a Bayesian phylodynamic analysis to reconstruct a time-resolved phylogeny using all the Nord Kivu outbreak EBOV genomes with at least 95% coverage (**Figure 2A**). We determined the overall rate of evolution for the EBOV Ituri-variant in the ongoing Nord Kivu outbreak to be 0.77E-3 subs/site/year, (95% HPD: 0.66E-3 - 0.88E-3), consistent with intra-outbreak rates observed from the 2013-2016 West African epidemic.^9^ The branch leading to d171 had a reduced rate of 0.21E-3 subs/site/year (95% HPD: 0.07E-3 - 0.38E-3, **Figure 2B** **and Figure S2**), a slowing of the molecular clock consistent with persistent EBOV infection.^10-13^ We found that the median estimated time to the most recent common ancestor (TMRCA) of all relapse clade genomes was November 7, 2019 (95% HPD: 15 Oct 2019 - 24 Nov 2019), which is consistent with recurrence of symptoms in the patient on November 25, 2019, and onward transmission shortly after. Taken together, our phylogenetic and epidemiological evidence shows that the patient’s second EVD episode was the result of EVD relapse from his initial EBOV infection, and not due to reinfection.

To investigate the potential failure of vaccine protection at his initial clinical presentation and relapse, we assayed the patient’s samples for anti-EBOV GP IgG antibody titers. We were unable to detect anti-EBOV GP IgG in sample d1, but we detected high titers 14 days later (d14) and in sample d171, eight days after the onset of relapse-associated illness (**Table 1****, Figure S3**). Based on the half-life of mAb114 (T1/2 ~ 24 days),^14^ the d14 results partially represent detection of residual mAb114. However, the patient would have cleared >99% of mAb114 when sample d171 was taken. The higher d171 titer likely resulted from the patient generating recall and/or primary antibody responses to the recurring infection. Thus, the explanation for relapse does not equate to a failed antibody response, raising the possibility that some other immune deficit (acquired or primary immune deficiency) may have played a role. Given the patient’s negative HIV test, we investigated the possibility of primary immune deficiencies to reconcile the patient’s course of disease and vaccine failure. We sequenced the patient’s exome, but no variants known or likely to cause primary immune deficiencies were detected (**Table S3**, **Figure S4)**.

While the location of the mutations in the viral envelope glycoprotein (GP) (E258K and E280G) are distant from the mAb114 binding site,^7^ it is reasonable to consider whether these mutations resulted from viral escape from mAb114 treatment. To test for this possibility, we evaluated the ability of mAb114 to neutralize viruses bearing the mutant GPs (**Table 1****, Figure S5**). The data showed nearly identical neutralization by mAb114 of both wildtype Ituri and patient-mutated GP viruses, demonstrating that *in vitro* the GP variation was independent of mAb114 selective pressures.

## Discussion

This report represents a case of acute EVD relapse that has led to human-to-human transmission. We were able to sequence EBOV genomes from the patient’s first (d1) and second (d171) EVD episodes, demonstrating that they differ by only two mutations and, therefore, represents a relapse of his initial EBOV infection. Our time-aware phylodynamic analysis demonstrates that the virus evolved at a 4-fold slower rate between d1 and d171 compared to the overall outbreak, indicative of relapse from a persistent EBOV infection. Our median TMRCA for the relapse clade of November 7, 2019, estimates the earliest time point when the virus likely exited persistence and resumed a normal rate of replication. This date is in agreement with the recurrence of EVD symptoms on November 25 reported by the patient, assuming a 2-21 day pre-symptomatic incubation period.

While the underlying mechanism of relapse associated with EBOV persistence in convalescent patients remains unclear, the three documented cases of relapse (including the case reported here) all received antibody-based therapy as part of the treatment for their initial infection. The benefits of mAb-based therapy have been demonstrated by the PALM randomized controlled trial.^15^ Questions remain as to whether passive immunotherapy could, in rare instances, be associated with viral relapse as historically documented in Argentine hemorrhagic fever patients treated with convalescent plasma.^16^ Both previous EVD relapse cases were repatriated individuals who developed severe EVD and received aggressive supportive care along with convalescent plasma and experimental therapeutics.^3,17,18^ Both recovered and during convalescence developed organ-specific inflammatory syndromes (uveitis, meningoencephalitis) that required additional treatment.^3,4^ The major and very consequential distinction between our case and the previous relapse cases is the extent of onwards transmission. Our case was symptomatic in the community for eight days, visiting two health care centers without precautionary care, resulting in 29 directly-linked cases of EVD. Providing an unfortunate proof-of-principle in this report that EVD survivors with relapse syndromes can transmit EBOV similar to acute EVD patients.

To investigate why the patient was not protected from infection following his vaccination in December 2018, we tested his serum from his initial June EVD episode for anti-EBOV-GP IgG titers, but detected none (Table 1, **Figure S3**). Given that we found no signs of immune deficiencies, this likely represents an incident of temporal or complete vaccine-failure. This finding is in line with other studies that reported up to 10% of EVD patients had been fully vaccinated for at least 10 days prior to ETU admission.^15^ In addition, serology data from the Liberian PREVAIL trials with more than 700 rVSV-ZEBOV vaccinated participants, showed that ~20% of vaccinated individuals did not develop positive Ebola IgG binding titers one month following vaccination.^19,20^ Combined, these findings raise concerns about the true effectiveness of the rVSV-ZEBOV vaccine, which has been estimated to be 100% in the Guinean “Ebola ça suffit!” trial and 97.5% in the preliminary report by the WHO from its use in DRC.^21,22^

An alternative hypothesis for the patient’s lack of protection during relapse, despite his anamnestic response, is potential viral escape during persistence. The E280G GP mutation that developed during the patient’s EBOV persistence may have allowed a replication advantage or immune escape, but our data show that the mutated GPs retained sensitivity to the treatment antibody, mAb114 (**Figure S5**). Alternatively, the mutation may simply be coincidental and viral persistence arose by infection of an immune-privileged compartment. The other noted mutation in this patient is in a non-coding region and little is known about the impact of intragenic region on gene expression in Ebola virus. Further, our patient did not have overt evidence of chronic diseases associated with immunosuppression and our whole-exome sequencing analysis did not reveal genomic variants known or likely to cause primary immune deficiencies (**Figure S4, Table S3**), though primary immunodeficiency cannot be fully ruled out.

During the Nord Kivu outbreak in the DRC, the provision of effective EVD therapeutics and supportive care has helped more than a thousand patients to exit ETUs as survivors, aptly called “vainqueurs” (Fr. *victors*) in Nord Kivu.^15^ Despite the positive impact these countermeasures may have had on individual lives, the overall case fatality rate of ~66% is similar to those observed during prior outbreaks.^23^ This can be partially attributed to the fact that the outbreak occurred in a conflict zone, with frequent disruptions to all aspects of the outbreak response.^24,25^ This case report demonstrates the need for continued monitoring of vaccine and therapeutic interventions and the power of having locally available genomic capabilities to support the outbreak response. Relapse of EVD appears to be a rare event, however it needs to be recognized, like sexual transmission, as a mechanism for onward transmission from persistently infected individuals. More data are needed to understand the mechanism and risks factors of EVD relapse in order to prevent future transmission events and protect the patients, their families, and their communities.

## Disclosure

Disclosure forms provided by the authors are available with the full text of this article at NEJM.org.
