# Supplementary material for: Ebola Virus Transmission Initiated by Systemic Ebola Virus Disease Relapse
Source: N Engl J Med. Author manuscript; Available in PMC 2021 Apr 1. (PMC7888312; doi:10.1056/NEJMoa2024670)
Supplement: Supplementary file 1 [file NEJM-2021-2024670-s1.pdf]

Supplementary Appendix

Table of Contents

Title Page ... *2*

Methods ... *3*

Ethics statement ... 3

Authors contributions ... 3

Viral sequencing ... 3

Viral sequencing analysis ... 3

EBOV IgG ELISAs ... 4

Human exome sequencing and analysis ... 4

Supplementary Figures and Tables: ... *6*

Supplementary Table S1: Blood chemistry Data ... 6

Supplementary Table S2: Sample metadata ... 7

Supplementary Table S3: Exome variants potentially linked to primary immune ... 9

deficiencies ... 9

Figure S1: Maximum Likelihood tree of 297 genomes from the current Nord-Kivu EBOV outbreak in DRCA) ... 10

Figure S2: Comparison of evolutionary rates between persistent infection branches and the overall outbreak. ... 11

Supplementary Figure S3: Anti-Ebola GP IgG ELISA data. ... 12

Supplementary Figure S4: Exome pathogenic variant analysis summary. ... 13

Supplementary Figure S5: mAb114 neutralization capacity against patient's GP mutants. 14

References ... *15*

Ebola virus transmission initiated by systemic Ebola virus disease relapse

Placide Mbala-Kingebeni[1,2*^], M.D., Ph.D Catherine Pratt[3*], M.Sc., Mbusa Mutafali Ruffin[4*], M.D., Matthias G. Pauthner[5*], Ph.D., Faustin Bile[6*], M.D., Antoine Nkuba Ndaye[1,2*], M.D, Allison Black[7*], Ph.D., Eddy Kinganda Lusamaki[1], M.D., Martin Faye[8], Ph.D., Amuri Aziza[1], M.Sc., Moussa M Diagne[8], Ph.D., Daniel Mukadi[1], M.D., Bailey White[3], B.Sc. James Hadfield[7], Ph.D., Karthik Gangavarapu[5], B.Pharm, Nella Bisento[1], M.Sc., Donatien Kazadi[1], M.D., Bibiche Nsunda[1], B.Sc., Marceline Akonga[1], B.Sc., Olivier Tshiani[1], M.D., John Misasi[9], M.D., Aurelie Ploquin[9], Ph.D., Victor Epaso[6], M.D., Emilia Sana Paka[6], M.D., Yannick Tutu Tshia N'kasar[6], M.D., Fabrice Mambu[2], M.D., Francois Edidi[2], M.D., Meris Matondo[2], M.D., Junior Bula Bula[2], M.D., Boubacar Diallo[10], M.D., Mory Keita[10], M.D., Marie Roseline Darnycka Belizaire[10], M.D., Ibrahima Soce Fall[10], Ph.D., Abdoulaye Yam[10], M.D., Mulangu Sabue[1], M.D., Anne W. Rimion[11], Ph.D., MPH, Elias Salfati[5], Ph.D., Ali Torkamani[5], Ph.D., Marc A. Suchard[11], M.D, Ph.D., Ian Crozier[12], M.D., Lisa Hensley[13], Ph.D., Andrew Rambaut[14], Ph.D., Ousmane Faye[8], Ph.D., Amadou Sall[8], Ph.D., Nancy J. Sullivan[9#], Ph.D., Trevor Bedford[7#], Ph.D., Kristian G. Andersen[5#], Ph.D., Michael R. Wiley[3#^], Ph.D., Steve Ahuka-Mundeke[1,2#], M.D., Ph.D., Jean-Jacques Muyembe Tamfum[1,2#], M.D., Ph.D.

[1] Institut National de Recherche Biomédicale, Kinshasa, DRC
[2]University of Kinshasa, Kinshasa, DRC
[3] University of Nebraska Medical Center, Omaha, NE, USA
[4] International Medical Corps, Los Angeles, CA, USA
[5] The Scripps Research Institute, La Jolla, CA, USA
[6] Ministère de la Sante, Kinshasa, DRC
[7] Fred Hutchinson Cancer Research Center, Seattle, WA, USA
[8] Institut Pasteur de Dakar, Dakar, Senegal
[9] Vaccine Research Center, National Institute of Allergy and Infectious Diseases (NIAID), National Institutes of Health (NIH), Bethesda, Maryland, USA
[10] World Health Organization, Geneva, CHE
[11] University of California, Los Angeles, CA, USA
[12] Clinical Monitoring Research Program Directorate, Frederick National Laboratory for Cancer Research, Frederick, MD, USA
[13] Integrated Research Facility at Fort Detrick, National Institute of Allergy and Infectious Diseases, National Institutes of Health, Frederick, MD, USA
[14] University of Edinburgh, Edinburgh, UK

*Authors contributed equally to this article, #co-senior authors
^corresponding authors: Placide Mbala-Kingebeni (mbalaplacide@gmail.com); Michael R. Wiley (mike.wiley@unmc.edu)

Methods

Ethics statement

The use of mAb114 during the June 2019 treatment was conducted under the Monitored Emergency Use of Unregistered and Investigational Interventions (MEURI) protocol which was review by Institut National de Recherche Biomédicale internal review board (IRB). The Ministry of Health (MoH) of the Democratic Republic of the Congo (DRC) approved this study. Oral consent was obtained at the homes of patients or in the Ebola Treatment Units prior to any sample collection by a team, including staff members of the MoH.

The sequencing and analysis of de-identified human diagnostic surveillance samples analyzed in this outbreak response project were also reviewed for the applicability of human subjects protection regulations by IRBs from Scripps and UNMC and was determined to not be human subject research.

Authors contributions

PMK, CP, MMR, MGP, TB, NJS, KGA, MRW, SAM, and JJMT designed the study, PMK, CP, MMR, MGP, FB, ANN, EKL, MF, AA, MMD, DM, BW, NB, DK, BN, MA, OT, AP, VE, ESP, YTTN, FM, FE, MM, JBB, BD, MK, MRDB, ISF, AY, MS, AWR, OF, and AS gathered the data, CP, MGP, AB, EKL, JH, KG, JM, ES, AT, MAS, IC, LH, AR, NJS, TB, KGA, and MRW analyzed the data, all authors vouch for the data and the analysis, PMK, CP, MMR, MGP, FB, ANN, AB, JH, KG, JM, IC, LH, TB, KGA, and MRW wrote the paper, and all authors listed in by-line decided to publish the paper. PMK, CP, MMR, MGP, AB, TB, KGA, and MRW wrote the first draft of our manuscript.

Viral sequencing

RNA was extracted from serum samples using the Qiagen Viral RNA Mini kit. cDNA was prepared using the ThermoFisher 1st strand synthesis system, and amplicons prepared using Q5 mastermix (New England Biolabs) and EBOV-specific amplicons generated using PrimalSeq.[1] Amplicons were quantified using a Qubit fluorometric quantification device (ThermoFisher) with dsDNA broad range kit and diluted to <500 ng for input into library preparation. Libraries were prepared using the Illumina Nextera DNA Flex kit with IDT for Illumina Unique Dual indexes, quantified, and loaded on the Illumina iSeq 100 for 2 x 150 cycles or Illumina MiSeq for 2 x 150 cycles.

Viral sequencing analysis

Short read data were analyzed with the iVar (v1.0.1)[2] using reference sequence MK007330. The reads were trimmed using the ivar trim command with a quality threshold of 20 and with option -e, to prevent the removal of reads that are not directly attached to amplicon primers following Nextera Flex library prep. Consensus sequences were called using the ivar consensus command,

and a minimum coverage depth threshold of 50x, to prevent inadvertent contamination. Negative controls were included in each run to monitor potential contamination.

Multiple sequence alignment was performed with MAFFT[3] after retaining only genomes that were >95% complete. The maximum likelihood analysis was performed using IQ-TREE using ModelFinder to select the best-fit model.[4] The Nextstrain phylogenetics platform was used as part of the analysis.[5] Bayesian phylogenetic analysis was performed using BEAST v1.10.5 to infer time-resolved phylogenies.[6] We used an SDR06 nucleotide substitution model with a local clock model and a non-informative continuous time Markov chain reference prior (CTMC) on the molecular clock rate and a Skygrid coalescent prior. All the Bayesian analyses were run for 80 million Markov chain Monte Carlo steps, sampling parameters and trees every 10,000 generations.[7,8] Tracer v1.7 was used to ensure run convergence (effective sample size > 200).[9] The BEAST XML and log files are available at https://github.com/andersen-lab/paper_2020_drc-ebola. The final figures were created using baltic (https://github.com/evogytis/baltic) and Phylo (https://biopython.org/wiki/Phylo).

EBOV IgG ELISAs

Anti-human Ebola GP IgG ELISA data was generated using the respective Alpha Diagnostic International kits, according to manufacturer's instructions. In brief, patient serum was diluted as indicated. Final incubation with TMB substrate was carried out for 15 minutes, before stopping solution was added. ELISA Plates were read at 450nm, and optical density at 630nm was subtracted to normalize well background. To analyze the data, blank background signal was subtracted from all data. EC_{50} binding titers were determined using Graphpad Prism 8.

Human exome sequencing and analysis

Human genomic DNA was isolated from blood using the Qiagen DNA Blood and Tissue kit. Exome sequencing libraries were prepared using the Illumina Nextera Flex for Enrichment kit, utilizing the Illumina Exome Panel as enrichment oligos and IDT for Illumina Unique Dual indexes according to manufacturer's instructions. Sequencing libraries were analyzed on an Agilent TapeStation using the high-sensitivity DNA reagents before dilution and loading. Two exome libraries were prepared from two independent DNA extractions of the same sample, to maximize exome coverage. Both libraries were loaded on a single Illumina MiSeq run, using 2 x 300 cycle V3 chemistry to maximize the coverage depth. Close to 20 million unique reads were obtained, resulting in an average depth of 69X across all variants found. Sequence quality control was done using the FastQC software package (https://www.bioinformatics.babraham.ac.uk/projects/fastqc/), and sequences were mapped to *hg19* using the BWA aligner.[10] SNP and INDEL calling, annotation, classification and in-depth analyses were carried out with the Genoox platform (https://www.genoox.com/). The analysis focused on genes causing immunodeficiency disorders. Upon analysis of likely pathogenic mutations using a built-in tool in the Genoox platform we identified a single likely pathogenic variant, c.356_357insC (p.Glu119fs), in the *SH2D1A* gene (Supplementary Table 2). This variant

has been reported to cause Lymphoproliferative Syndrome, OMIM # 308240.[11] However, Sanger sequencing confirmation revealed that this variant arose from a sequencing error, and was in fact not present in the patient's genome.

Neutralization assay

mAb114 recognition of patient EBOV GP mutants was evaluated using a single-round infection and neutralization assay. Lentiviruses were produced bearing at their surface EBOV GP from either Ituri wildtype GP (first sequenced virus 18FHV089[12]), patient's first infection sequenced GP (d1, MAN4194) and patient's second infection sequenced GP (d171, MAN12309). Neutralization was performed as previously described.[13] Briefly, HEK293T cells were exposed to pseudovirus mixed with serial dilutions of mAb114 at concentrations from 0.0001–10 µg/mL. Luciferase activity measured as relative luminescence unit (RLU) was obtained after lysis of target cells by using a Luciferase Assay System Bright Glo (Promega) and an Envision Plate Reader (PerkinElmer). Assays were performed three times, each with samples in triplicate.

Supplementary Figures and Tables:

Supplementary Table S1: Blood chemistry Data

Blood chemistry data, as measured by the Piccolo Xpress system (Abaxis) on December 4 during the second Ebola episode. The results indicate multiple organ failure and the patient passed away the same day.

Test	Result	Normal range
Glycemia	126 mg/dl	73-118
BUN	131 mg/dl	7-22
CRE	7.6 mg/dl	0.6-1.2
TBIL	5.4 mg / dl	0.2-1.6
ALB	1.5 g/dl	3.3-5.5
ALT	431 U/l	10-47
AST	1221 U/l	11-38
CK	1400 U/l	30-380
AMY	436 U/l	14-97
Na^+	126 mmol/l	128-145
K^+	4.0 mmol/l	3.6-5.1
CA	7.0 mg/dl	8.0-10.3
CRP	169 mg/l	0-7.5

Supplementary Table S2: Sample metadata

Lab ID	Date sample tested	Health zone	Province	Genome coverage	Date sample sequenced
MAN4194	16-Jun-19	Mabalako	Nord-Kivu	99.58%	20-Dec-19
MAN12309	3-Dec-19	Mabalako	Nord-Kivu	96.67%	20-Dec-19
MAN12369	5-Dec-19	Mabalako	Nord-Kivu	99.65%	23-Jan-20
MAN12448	7-Dec-2019	Mabalako	Nord-Kivu	99.70	17-Dec-19
MAN12460	8-Dec-19	Mabalako	Nord-Kivu	99.99%	17-Dec-19
MAN12468	8-Dec-19	Mabalako	Nord-Kivu	98.55%	17-Dec-19
MAN12470	8-Dec-19	Mabalako	Nord-Kivu	99.97%	17-Dec-19
MAN12472	8-Dec-19	Mabalako	Nord-Kivu	99.23%	17-Dec-19
MAN12506	10-Dec-19	Mabalako	Nord-Kivu	99.22%	17-Dec-19
MAN12508	10-Dec-19	Mabalako	Nord-Kivu	98.41%	17-Dec-19
MAN12514	10-Dec-2019	Mabalako	Nord-Kivu	98.52	17-Dec-19
MAN12535	10-Dec-19	Mabalako	Nord-Kivu	98.31%	17-Dec-19
MAN12541	10-Dec-19	Mabalako	Nord-Kivu	99.87%	17-Dec-19
MAN12542	10-Dec-19	Mabalako	Nord-Kivu	99.56%	17-Dec-19
MAN12545	10-Dec-19	Mabalako	Nord-Kivu	95.52%	17-Dec-19
MAN12546	10-Dec-19	Mabalako	Nord-Kivu	99.97%	17-Dec-19
MAN12581	11-Dec-19	Mabalako	Nord-Kivu	99.97%	17-Dec-19
MAN12589	11-Dec-19	Biena	Nord-Kivu	98.91%	17-Dec-19
MAN12727	13-Dec-19	Mabalako	Nord-Kivu	99.85%	23-Jan-20
MAN12770	14-Dec-19	Mabalako	Nord-Kivu	99.87%	23-Jan-20
MAN12790	14-Dec-19	Mabalako	Nord-Kivu	99.85%	23-Jan-20
BTB39991	17-Dec-19	Butembo	Nord-Kivu	92.10%	10-Jan-20
MAN12952	17-Dec-19	Mabalako	Nord-Kivu	99.73%	21-Jan-20
MAN12990	18-Dec-19	Mabalako	Nord-Kivu	99.66%	21-Jan-20
MAN12999	18-Dec-19	Mabalako	Nord-Kivu	99.85%	27-Feb-20
MAN13030	19-Dec-19	Mabalako	Nord-Kivu	99.27%	27-Feb-20
MAN13175	21-Dec-19	Mabalako	Nord-Kivu	99.99%	2-Mar-20
MAN13221	22-Dec-19	Mabalako	Nord-Kivu	96.76%	23-Jan-20
MAN13222	22-Dec-19	Mabalako	Nord-Kivu	99.79%	21-Jan-20
MAN13238	22-Dec-19	Mabalako	Nord-Kivu	99.91%	23-Jan-20
MAN13273	23-Dec-19	Mabalako	Nord-Kivu	99.89%	11-Jan-20
MAN13347	24-Dec-19	Mabalako	Nord-Kivu	99.88%	21-Jan-20
BTB41146	25-Dec-19	Butembo	Nord-Kivu	99.91%	11-Jan-20
MAN13348	25-Dec-19	Mabalako	Nord-Kivu	99.88%	23-Jan-20
MAN13384	25-Dec-19	Mabalako	Nord-Kivu	99.91%	21-Jan-20
KAT21808	31-Dec-19	Butembo	Nord-Kivu	88.81%	11-Jan-20

MAN13677	31-Dec-19	Mabalako	Nord-Kivu	99.90%	27-Feb-20
BTB42007	1-Jan-20	Musienene	Nord-Kivu	98.06%	11-Jan-20
KAT21874	1-Jan-20	Butembo	Nord-Kivu	99.63%	10-Jan-20
BTB42105	2-Jan-20	Butembo	Nord-Kivu	87.95%	11-Jan-20
BTB42106	2-Jan-20	Butembo	Nord-Kivu	99.65%	11-Jan-20
BTB42291	3-Jan-20	Butembo	Nord-Kivu	98.34%	11-Jan-20
MAN13942	6-Jan-20	Mabalako	Nord-Kivu	98.09%	27-Jan-20
BEN45861	7-Jan-20	Beni	Nord-Kivu	92.61%	30-Jan-20
BEN46116	9-Jan-20	Beni	Nord-Kivu	95.74%	30-Jan-20
KAT22664	11-Jan-20	Butembo	Nord-Kivu	93.12%	27-Feb-20
KAT22674	11-Jan-20	Mabalako	Nord-Kivu	95.82%	27-Jan-20
MAN14198	12-Jan-20	Mabalako	Nord-Kivu	95.48%	21-Jan-20
BEN46488	13-Jan-20	Beni	Nord-Kivu	99.04%	2-Mar-20
MAN14228	13-Jan-20	Mabalako	Nord-Kivu	99.73%	5-Feb-20
MAN14231	13-Jan-20	Mabalako	Nord-Kivu	93.98%	30-Jan-20
MAN14242	13-Jan-20	Mabalako	Nord-Kivu	99.44%	28-Feb-20
MAN14243	13-Jan-20	Mabalako	Nord-Kivu	92.46%	30-Jan-20
BEN46574	14-Jan-20	Beni	Nord-Kivu	86.10%	30-Jan-20
MAN14263	14-Jan-20	Mabalako	Nord-Kivu	92.60%	30-Jan-20
MAN14285	14-Jan-20	Mabalako	Nord-Kivu	99.89%	12-Feb-20
BEN46697	15-Jan-20	Beni	Nord-Kivu	99.86%	3-Feb-20
BEN46737	15-Jan-20	Beni	Nord-Kivu	99.59%	28-Feb-20
BEN46754	15-Jan-20	Beni	Nord-Kivu	99.89%	28-Feb-20
BEN46848	16-Jan-20	Beni	Nord-Kivu	98.98%	12-Feb-20
BEN47083	18-Jan-20	Beni	Nord-Kivu	99.91%	27-Feb-20
BEN47206	19-Jan-20	Beni	Nord-Kivu	99.84%	27-Jan-20
BEN47225	19-Jan-20	Beni	Nord-Kivu	99.94%	27-Feb-20
BEN47296	20-Jan-20	Beni	Nord-Kivu	99.91%	27-Feb-20
BEN47371	20-Jan-20	Beni	Nord-Kivu	97.55%	27-Feb-20
BEN48039	25-Jan-20	Beni	Nord-Kivu	99.85%	5-Feb-20
BEN48100	26-Jan-20	Beni	Nord-Kivu	99.85%	3-Feb-20
BEN48231	27-Jan-20	Beni	Nord-Kivu	99.89%	5-Feb-20
BEN48404	28-Jan-20	Beni	Nord-Kivu	99.92%	5-Feb-20
BEN48608	29-Jan-20	Beni	Nord-Kivu	99.96%	5-Feb-20
BEN49243	4-Feb-20	Beni	Nord-Kivu	95.40%	20-Feb-20
BEN49411	5-Feb-20	Beni	Nord-Kivu	87.39%	12-Feb-20
BEN49660	7-Feb-20	Beni	Nord-Kivu	87.01%	12-Feb-20
BEN50212	11-Feb-20	Beni	Nord-Kivu	97.20%	20-Feb-20
BEN50970	17-Feb-20	Beni	Nord-Kivu	96.29%	1-Mar-20

Supplementary Table S3: Exome variants potentially linked to primary immune deficiencies

Gene	Variation Type	Chr	Start Position	Stop Position	Ref	Alt	Transcript	AA Change	Nucleotide	Exon	Zygosity	Region	Effect	Confidence	Quality	Genotype (	Genotype L	Depth	Ref Depth	Obs Depth	Genoox Classification
CFTR	SNP	chr7	117306984	117306984	G	A	NM_000492.3	p.Arg1422Gln	c.4265G>A	27	het	Exonic	Missense	High	927.77	99	956:0:1614	135	80	55	Uncertain - Possibly Pathogenic (Low)
CIITA	SNP	chr16	10992836	10992836	T	C	NM_000246.3	p.Val138Ala	c.413T>C	5	het	Exonic	Missense	High	876.77	99	905:0:2275	172	114	58	Uncertain - Possibly Pathogenic (Low)
SH2D1A	Indel	chrX	123505210	123505210	A	AC	NM_002351.4	p.Glu119fs	c.356_357insC	4	het	Exonic	Frameshift	Low	13.9273	14		13	10	3	Likely Pathogenic
GHR	SNP	chr5	42713631	42713631	G	T	NM_000163.5		c.875+10G>T	8	het	Splice Region		Medium	137.77	99	166:0:380	27	17	10	Uncertain Significance
GHR	SNP	chr5	42718592	42718592	T	C	NM_000163.5	p.Ile328Thr	c.983T>C	10	het	Exonic	Missense	High	502.77	99	531:0:466	54	26	28	Uncertain Significance
XIAP	SNP	chrX	123025117	123025117	A	T	NM_001167.3	p.Gln336Leu	c.1007A>T	4	het	Exonic	Missense	Low	10.6282	11		10	8	2	Uncertain Significance
SDHB	SNP	chr1	17354329	17354329	G	A	NM_003000.2	p.Ser152Phe	c.455C>T	5	het	Exonic	Missense	High	902.77	99	931:0:1583	135	81	54	Uncertain - Possibly Pathogenic (Low)
C5	SNP	chr9	123737151	123737151	C	T	NM_001735.2	p.Arg1308His	c.3923G>A	30	het	Exonic	Missense	High	620.77	99	649:0:550	64	30	34	Uncertain Significance
OFD1	SNP	chrX	13774707	13774707	A	T	NM_003611.3	p.Glu411Val	c.1232A>T	13	het	Exonic	Missense	Low	23.842	21		4	2	2	Uncertain - Possibly Pathogenic (Low)

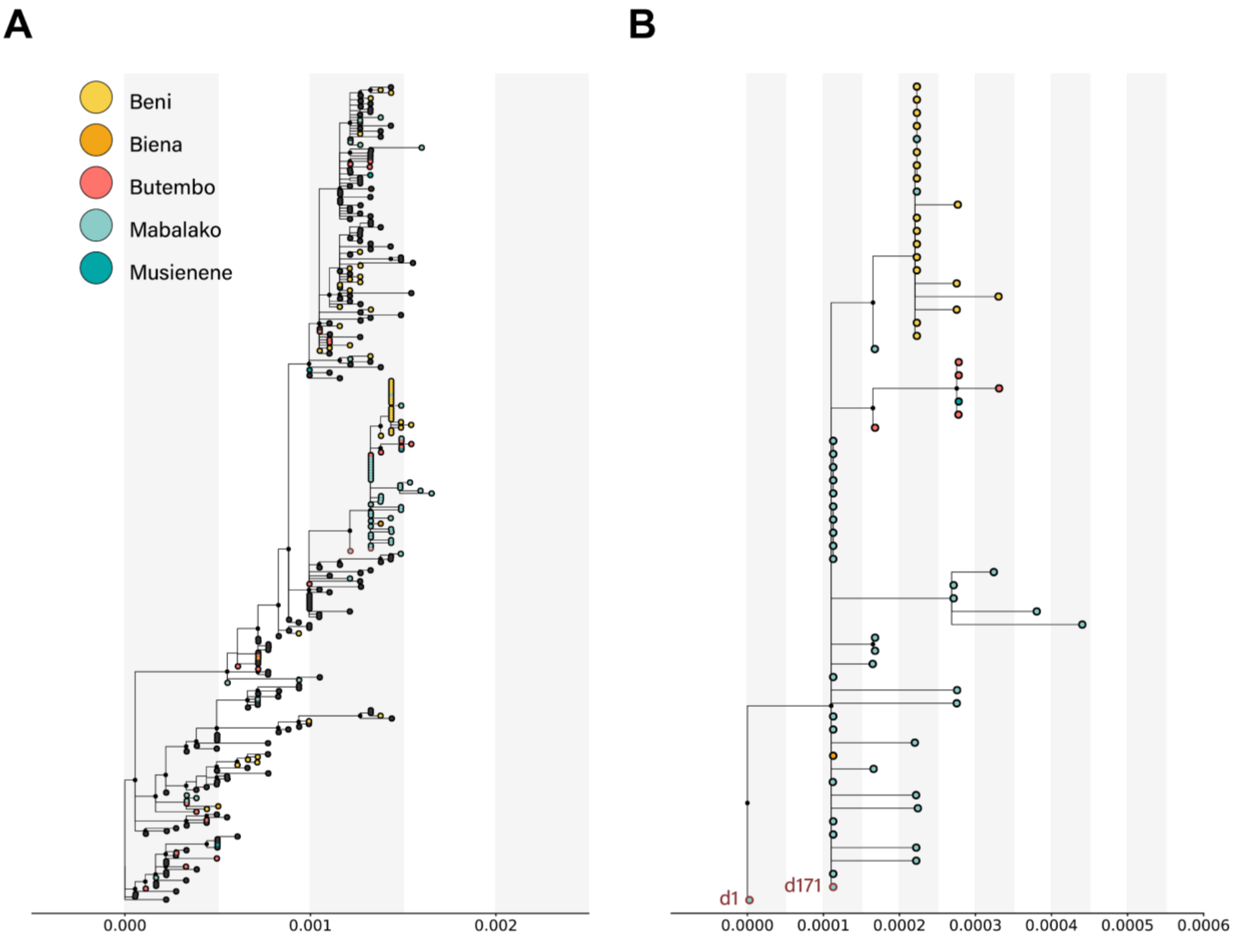

Figure S1: Maximum Likelihood tree of 297 genomes from the current Nord-Kivu EBOV outbreak in DRCA)

Maximum Likelihood (ML) tree of sequenced isolates from the current EBOV outbreak in DRC (n=297), colored by health zones relevant to this study as indicated. B) Zoomed in view of the ML tree showing the first (d1) and second EVD episodes (d171) of the relapse patient, as well as 61 viral genomes sampled from epidemiologically linked cases. Two mutations (T5578C - non coding, A6867G - GP E280G) developed during the persistent infection of the relapse patient and are unique to Sample d171 and the 61 samples from the relapse cluster, showing human-to-human transmission originating from the relapse patient. The horizontal axes show the number of nucleotide substitutions per site. Data taken from https://nextstrain.org/community/inrb-drc/ebola-nord-kivu and released on NCBI GenBank database.

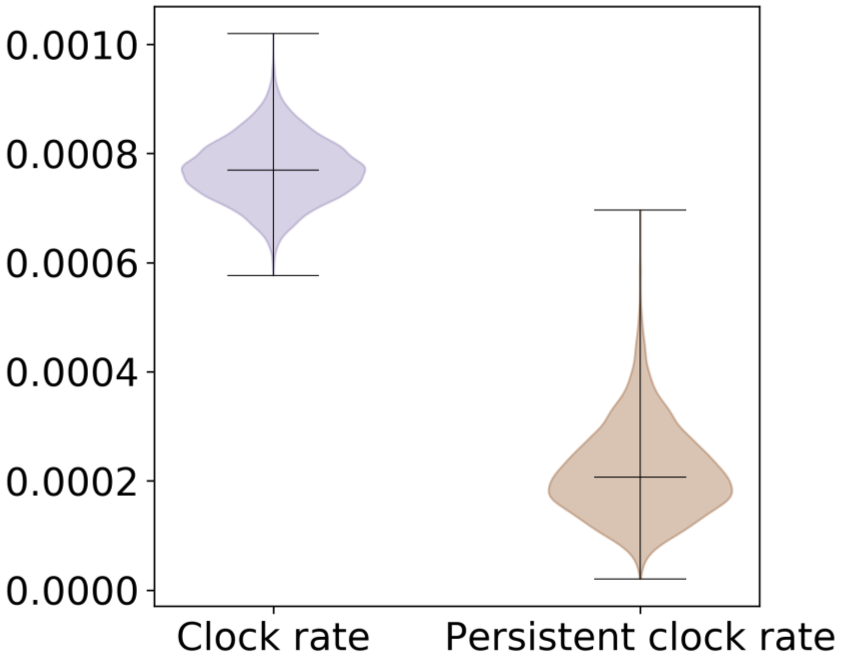

Figure S2: Comparison of evolutionary rates between persistent infection branches and the overall outbreak.

Based on preliminary Bayesian phylogenetic analysis using Beast with a relaxed clock model, we allowed three branches originating from samples d1, KAT21596 and MAN14985, which are strongly presumed to represent persistent infections, to have a different evolutionary rate from the rest of the tree, under a local clock model. This allows for the comparison of persistent infection and overall outbreak evolutionary clock rates. The median evolutionary rate for the overall outbreak was 0.00077 substitutions/site/year (clock rate; 95% HPD: 0.00066 - 0.00088), while the median persistent evolutionary rate was approximately 4-fold lower at 0.000207 substitutions/site/year (persistent clock rate; 95% HPD: 0.00007, 0.00038). Shown are posterior density distributions with median and the range indicated by black lines.

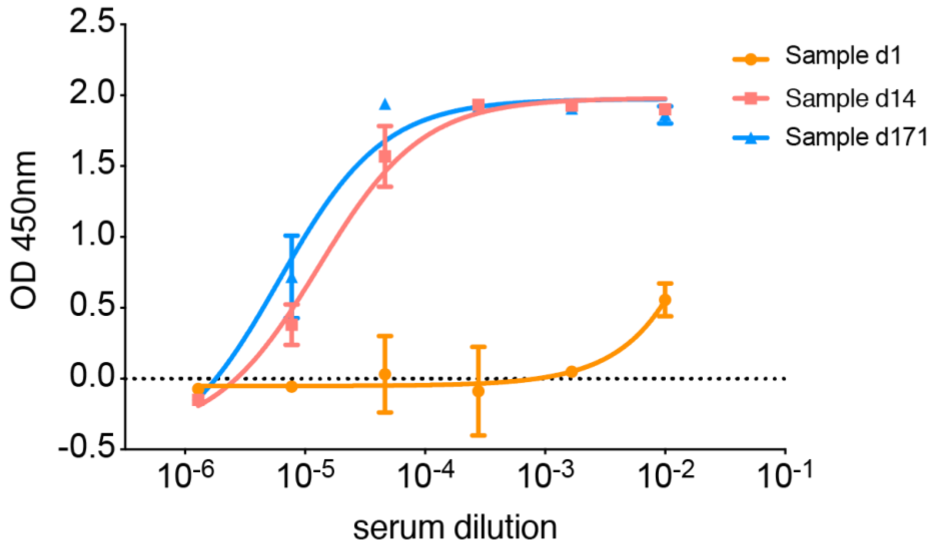

Supplementary Figure S3: Anti-Ebola GP IgG ELISA data.

Anti- Ebola GP IgG ELISA results of serial serum dilutions from the first and second EVD episodes. Titers were calculated using Graphpad Prism 8 and are listed in Table 1.

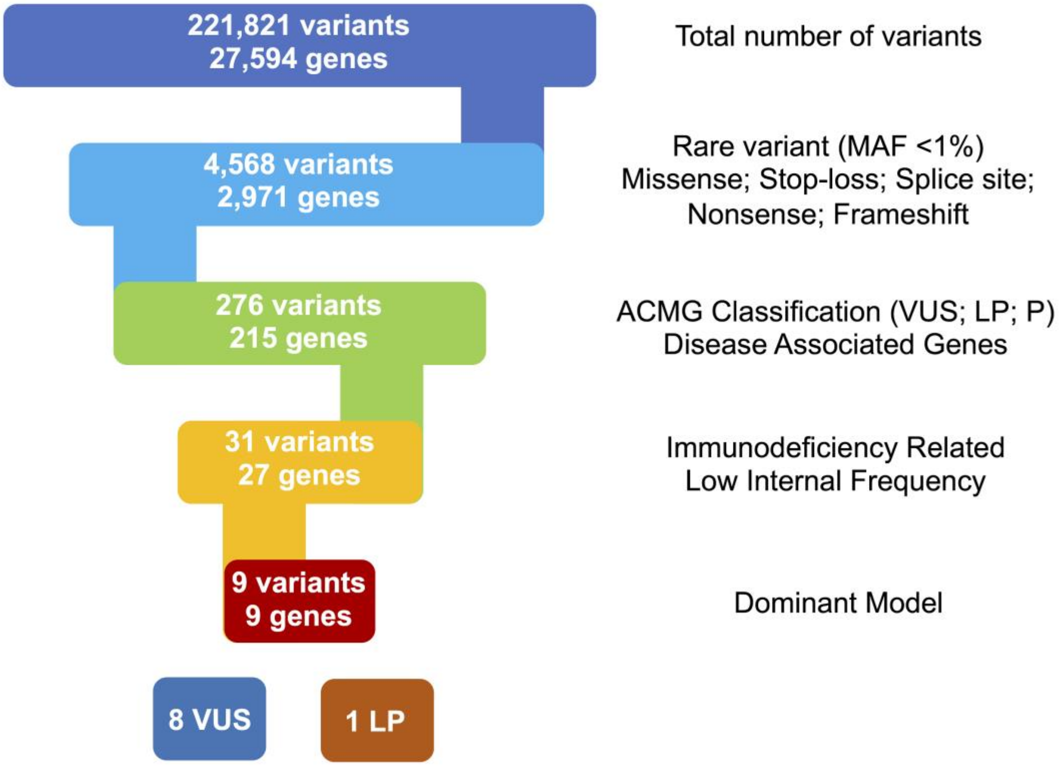

Supplementary Figure S4: Exome pathogenic variant analysis summary.

Over 20 million exome reads were aligned to the human reference genome, yielding an average variant coverage of 69-fold across all 221,821 screened variants. The analysis was focused on variants that are potentially causing primary immune deficiencies, of which we found nine candidates in the filtered data set. Only one variant was found to be likely pathogenic, which we attempted to confirm with Sanger sequencing confirmation. However, the detected variant could be traced to a sequencing error. MAF: Minor Allele Frequency, ACMG: American College of Medical Genetics, VUS: Variant of Uncertain Significance, LP: Likely Pathogenic, P: Pathogenic.

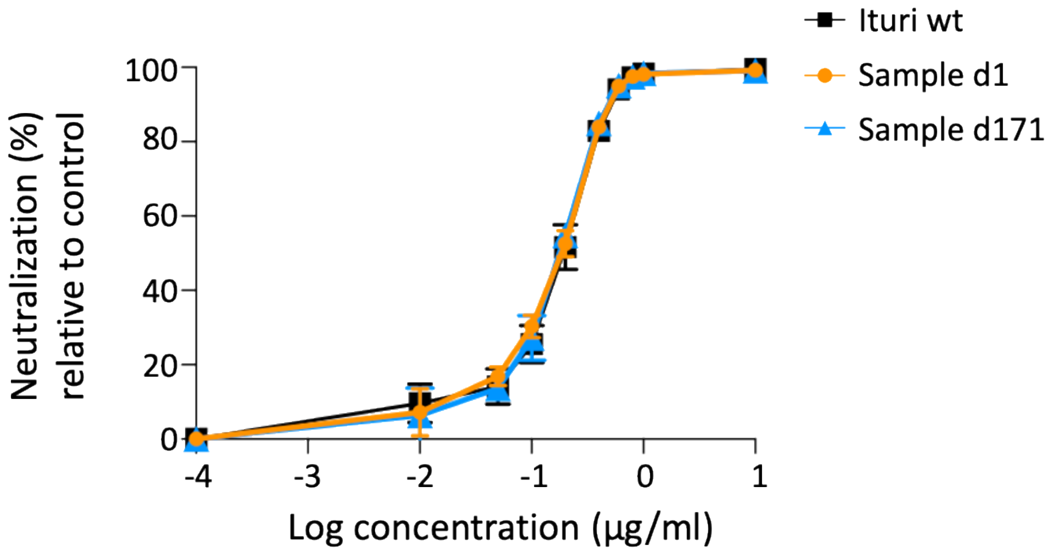

Supplementary Figure S5: mAb114 neutralization capacity against patient’s GP mutants.

Neutralization capacity of mAb114 was evaluated against pseudoparticles harboring either Ituri wildtype GP (18FHV089), patient’s first infection GP (d1, MAN4194) and patient’s second infection GP (d171, MAN12309).

References

1. Quick J, Grubaugh ND, Pullan ST, et al. Multiplex PCR method for MinION and Illumina sequencing of Zika and other virus genomes directly from clinical samples. Nat Protoc 2017;12:1261-76.
2. Grubaugh ND, Gangavarapu K, Quick J, et al. An amplicon-based sequencing framework for accurately measuring intrahost virus diversity using PrimalSeq and iVar. Genome Biol 2019;20:8.
3. Katoh K, Standley DM. MAFFT multiple sequence alignment software version 7: improvements in performance and usability. Mol Biol Evol 2013;30:772-80.
4. Nguyen LT, Schmidt HA, von Haeseler A, Minh BQ. IQ-TREE: a fast and effective stochastic algorithm for estimating maximum-likelihood phylogenies. Mol Biol Evol 2015;32:268-74.
5. Hadfield J, Megill C, Bell SM, et al. Nextstrain: real-time tracking of pathogen evolution. Bioinformatics 2018;34:4121-3.
6. Suchard MA, Lemey P, Baele G, Ayres DL, Drummond AJ, Rambaut A. Bayesian phylogenetic and phylodynamic data integration using BEAST 1.10. Virus Evol 2018;4:vey016.
7. Gill MS, Lemey P, Faria NR, Rambaut A, Shapiro B, Suchard MA. Improving Bayesian population dynamics inference: a coalescent-based model for multiple loci. Mol Biol Evol 2013;30:713-24.
8. Shapiro B, Rambaut A, Drummond AJ. Choosing appropriate substitution models for the phylogenetic analysis of protein-coding sequences. Mol Biol Evol 2006;23:7-9.
9. Rambaut A, Drummond AJ, Xie D, Baele G, Suchard MA. Posterior Summarization in Bayesian Phylogenetics Using Tracer 1.7. Syst Biol 2018;67:901-4.
10. Li H, Durbin R. Fast and accurate short read alignment with Burrows-Wheeler transform. Bioinformatics 2009;25:1754-60.
11. Purtilo DT, Sakamoto K, Barnabei V, et al. Epstein-Barr virus-induced diseases in boys with the X-linked lymphoproliferative syndrome (XLP): update on studies of the registry. Am J Med 1982;73:49-56.
12. Mbala-Kingebeni P, Aziza A, Di Paola N, et al. Medical countermeasures during the 2018 Ebola virus disease outbreak in the North Kivu and Ituri Provinces of the Democratic Republic of the Congo: a rapid genomic assessment. Lancet Infect Dis 2019;19:648-57.
13. Corti D, Misasi J, Mulangu S, et al. Protective monotherapy against lethal Ebola virus infection by a potently neutralizing antibody. Science 2016;351:1339-42.
